# Therapeutic Strategies to Reduce the Toxicity of Misfolded Protein Oligomers

**DOI:** 10.3390/ijms21228651

**Published:** 2020-11-17

**Authors:** Ryan P. Kreiser, Aidan K. Wright, Natalie R. Block, Jared E. Hollows, Lam T. Nguyen, Kathleen LeForte, Benedetta Mannini, Michele Vendruscolo, Ryan Limbocker

**Affiliations:** 1 Department of Chemistry and Life Science, United States Military Academy, West Point, NY 10996, USA; ryan.kreiser@westpoint.edu (R.P.K.); aidan.wright@westpoint.edu (A.K.W.); natalie.block@westpoint.edu (N.R.B.); jared.hollows@westpoint.edu (J.E.H.); lam.nguyen@westpoint.edu (L.T.N.); kathleen.leforte@westpoint.edu (K.L.); 2 Centre for Misfolding Diseases, Department of Chemistry, University of Cambridge, Cambridge CB2 1EW, UK; bm475@cam.ac.uk

**Keywords:** misfolded protein oligomers, countermeasures, kinetics, structure–toxicity relationships, membrane protection, protein homeostasis, Alzheimer’s disease, Parkinson’s disease

## Abstract

The aberrant aggregation of proteins is implicated in the onset and pathogenesis of a wide range of neurodegenerative disorders, including Alzheimer’s and Parkinson’s diseases. Mounting evidence indicates that misfolded protein oligomers produced as intermediates in the aggregation process are potent neurotoxic agents in these diseases. Because of the transient and heterogeneous nature of these elusive aggregates, however, it has proven challenging to develop therapeutics that can effectively target them. Here, we review approaches aimed at reducing oligomer toxicity, including (1) modulating the oligomer populations (e.g., by altering the kinetics of aggregation by inhibiting, enhancing, or redirecting the process), (2) modulating the oligomer properties (e.g., through the size–hydrophobicity–toxicity relationship), (3) modulating the oligomer interactions (e.g., by protecting cell membranes by displacing oligomers), and (4) reducing oligomer toxicity by potentiating the protein homeostasis system. We analyze examples of these complementary approaches, which may lead to the development of compounds capable of preventing or treating neurodegenerative disorders associated with protein aggregation.

## 1. Protein Aggregation and Its Links with Neurodegenerative Diseases

A wide variety of human disorders, including Alzheimer’s disease (AD) and other forms of dementia, are associated with ageing and unhealthy lifestyles [1]. These diseases are still largely incurable and represent a terrible burden for our societies and healthcare systems [2,3]. Over 50 million people currently suffer from dementia worldwide, a number estimated to triple within the next three decades unless effective treatments become available [4]. These conditions are characterized by the presence of aberrant proteinaceous deposits in affected tissues [5,6,7,8,9,10].

To understand the molecular origins of these widespread disorders, we highlight two observations. The first observation is about the generic nature of the phenomenon of protein misfolding and aggregation [11]. The amide and carbonyl groups of the backbone of the polypeptide chains of protein molecules have a strong tendency to form hydrogen bonds. Because the polypeptide backbone is common to all proteins independent of their amino acid sequences, under suitable conditions, almost any protein can access the amyloid state [1,12]. While at low protein concentrations, hydrogen bonds form intra-molecularly, leading to the formation of protein secondary structures, such as α-helices and β-sheets that are characteristic of the native states of proteins, at high concentrations, the formation of inter-molecular hydrogen bonds is favored, so that proteins tend to self-assemble into a highly ordered structures known as amyloid fibrils, which are stabilized by a network of hydrogen bonds [12]. The second observation is that the cellular concentrations of proteins are typically close to the critical values that define whether the most stable state is the native or the amyloid state [13,14,15]. This observation, which has been referred to as “life at the edge of solubility” [13], has an evolutionary origin. Proteins that are highly soluble are subject to an evolutionary drift in which random mutations that are neutral in functional terms decrease the overall solubility of the proteins. This type of mutation goes unchallenged by natural selection until the solubility decreases to the critical level, beyond which negative selection prevents further reductions in solubility [13]. As a result, proteins tend to be supersaturated in the cellular environment and they are, therefore, on the edge of aggregation [16,17]. Although it is perhaps surprising, we now know that proteins commonly aggregate under physiological conditions [15], and it is only the presence of a strong protein homeostasis system that prevents the progressive accumulation of large protein deposits [12]. Overall, the natural tendency of proteins to aggregate when they are supersaturated predisposes certain cell and tissues to protein aggregation upon stress or ageing [18,19,20,21].

These observations lead to the conclusion that in order to prevent or treat protein misfolding diseases, one should find ways to pharmacologically augment or protect the protein homeostasis system that regulates protein aggregation. The pharmacological interventions that we review in the following sections are meant to address precisely this point.

## 2. Misfolded Protein Oligomers and Their Cytotoxic Effects

The abundance of amyloid deposits in the brains of patients afflicted by neurodegenerative diseases initially implicated mature amyloid fibrils as the central toxic agents. Similar deposits are observed for numerous other neurodegenerative diseases, including chronic traumatic encephalopathy, dementia with Lewy bodies, and Parkinson’s disease (PD). Although in this review we generally analyze the links between protein aggregation and human disease, we focus predominantly on the proteins associated with AD and PD, because they have been investigated more extensively. More recently, it has been realized that amyloid fibrils may not be directly responsible for toxic gain-of-function. Instead, transient, metastable oligomeric intermediates may be more neurotoxic [22]. An important role for these aggregates started to emerge over two decades ago, when pre-fibrillar species comprised of the 42-residue form of amyloid-β peptide (Aβ42), termed Aβ-derived diffusible ligands (ADDLs), were reported as potent neurotoxic species and shown to induce cellular death at nM concentrations [23]. Antibodies against ADDLs, but not monomeric or fibrillar Aβ, were found to interact with AD brain samples [24,25,26]. Soon afterwards, other soluble assemblies were similarly described as toxic forms of Aβ [27,28]. Many studies have since shown the presence of oligomers in the brain of patients affected by AD [29] and PD [30] and have reported that these aggregates are seminal in the induction of cellular dysfunction [31,32,33,34,35]. In a similar way, the onset of PD is characterized by the presence of Lewy bodies and Lewy neurites comprised of the α-synuclein protein (αS) in the brains of patients with the disease, and evidence indicates the source of neuronal death is the oligomeric phase of α-synuclein [36]. It is now well accepted that oligomers are key pathological species in protein misfolding diseases associated with amyloid deposition [7,31,37,38,39].

**Figure 1:**
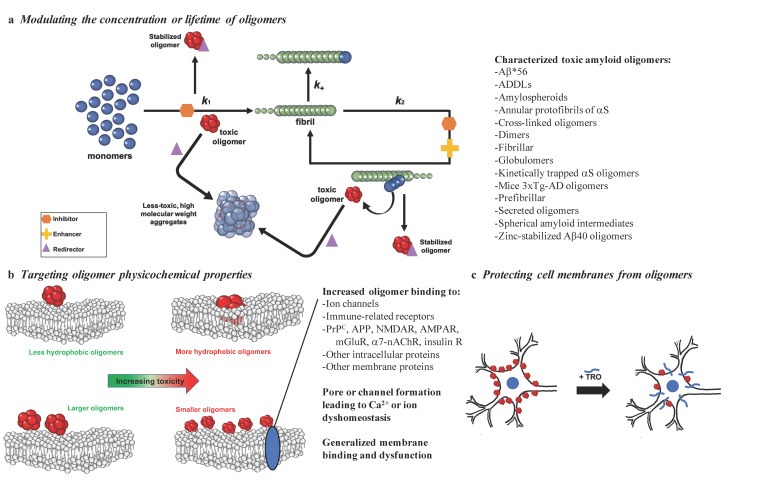
Possible strategies to target the toxicity of misfolded protein oligomers. With the goal of reducing the toxicity of misfolded protein oligomers, promising methods include: (**a**) targeting oligomer populations by inhibiting (orange), enhancing (yellow), or redirecting (purple) the protein aggregation reaction (*k*_1_, primary nucleation; *k*_2_, secondary nucleation; *k*_+_, elongation), (**b**) targeting the properties responsible for the ability of oligomers to induce cell membrane dysfunction by binding to specific receptors or generally to cell membranes, or (**c**) modifying cell membranes to prevent disruptive oligomer interactions, as exemplified by trodusquemine (TRO). A subset of the toxic oligomers that have been characterized thus far [23,29,32,40,41,42,43,44,45,46,47,48,49,50] are listed in (**a**) to illustrate the striking heterogeneity in their structures, physiochemical properties, and biological activities.

Misfolded protein oligomers can be formed as intermediates in the aggregation reaction of various amyloidogenic peptides or proteins from primary and secondary nucleation processes or from fragmentation of mature fibrils [37]. The heterogeneous nature of the oligomers that form during the aggregation process is reflected in the report of many different types of these assemblies, including Aβ*56 [32], ADDLs [23], amylospheroids [40], annular protofibrils of αS [41], dimers [29], fibrillar oligomers [42], covalently stabilized oligomers [43], globulomers [44], kinetically trapped αS oligomers [45], mice 3×Tg-AD oligomers [46], prefibrillar oligomers [47], secreted oligomers [48], spherical amyloid intermediates [49], and zinc-stabilized Aβ40 oligomers [50] (Figure 1a).

These species have been found to cause cellular dysfunction in a wide range of manners, including by interacting with the lipid and protein components of the cell [31], resulting in the disruption of homeostatic mechanisms and the induction of cell death [51,52]. Oligomeric species of disease-related aggregated proteins are highly toxic owing to their structural properties, such as small size and high hydrophobicity [53,54,55,56,57], which makes them highly diffusible and prone to interact with cell proteins and membranes. Notably, a direct relationship has been established between the cytotoxicity of amyloid aggregates and their molecular weight [57]. In particular, it has been illustrated that small A11 antibody-positive aggregates reduced the viability of cells by up to 80% in comparison to fibrils, which only reduced cellular health by approximately 20% [31].

A great deal of effort has therefore been devoted to the discovery of inhibitors of protein aggregation, which suppress oligomer generation through a variety of mechanisms [58,59,60,61,62,63,64], leading to reports of hundreds of compounds that can potentially reduce the rate of protein self-assembly. It has been much more difficult, however, to demonstrate the efficacy of these compounds in the clinical setting to become FDA-approved, disease-modifying therapeutics [3,65]. This situation can be attributed, in part, to the limited understanding of the mechanisms by which aggregation occurs, of its effects on the different cell types that comprise specific vulnerable tissues, of the means by which these compounds modify the aggregation process, and, critically, to their administration at too late a stage in a clinical situation where the amyloid load has already reached high levels [37].

In this review, we analyze recent strategies to suppress the toxicity linked to neurodegenerative diseases with a critical focus on demonstrated molecular agents that have shown the ability to combat the toxicity caused by oligomers. In particular, we assess species that (1) modulate populations of oligomers by perturbing their respective protein aggregation reactions via inhibiting, enhancing, or redirecting its reactive flux network (Figure 1a and Section 3), (2) target directly the properties of oligomers linked to their ability to induce cell damage (Figure 1b and Section 4), (3) protect cell membranes by targeting oligomer interactions (Figure 1c and Section 5), or (4) preserve the health of cells through all of the above by regulation of the protein homeostasis network (see Section 6). These approaches demonstrate examples of potential therapeutics designed to suppress the negative effects of toxic oligomeric assemblies, as well as highlight the diversity of mechanisms by which amyloid aggregation can be targeted. The hope is that future work using these promising lead strategies will culminate in the development of therapeutically relevant treatments that can arrest the devasting effects of neurodegeneration by targeting specifically misfolded protein oligomers.

## 3. Targeting the Kinetics of Formation of Misfolded Protein Oligomers

Due to the transient nature of the oligomers produced during the aggregation processes, many therapeutics rely on modulating the kinetics of oligomer assembly to reduce the populations of oligomers that are responsible for inducing cell death. We describe here three modes by which the kinetics can be altered using therapeutics, including aggregation inhibition, acceleration, or redirection, where inhibition reduces oligomer concentrations, acceleration shifts the reactive flux towards the final fibrillar form to reduce the lifetime of oligomers, and redirection leads to the formation of off-pathway species that are less toxic than on-pathway oligomeric aggregates (Figure 1a).

### 3.1. Inhibiting Protein Aggregation to Reduce the Number of Oligomeric Species

A series of recent studies on the modulation of the kinetics of Aβ and αS oligomer assembly has revealed that this type of intervention can suppress the cytotoxicity associated with protein aggregation [56,58,61,62,63,64,66,67,68]. While the exact mechanism of inhibition varies significantly for the diverse array of small molecules, antibodies, molecular chaperones, and other agents that have demonstrated efficacy in delaying the aggregation process, the biological importance of inhibition is that the inhibitor prevents oligomer formation to reduce their populations by preventing some step in the aggregation process of their respective oligomer assembly reaction. Additionally, some of these molecules can not only interrupt the formation of oligomer aggregates, but also induce the disaggregation of toxic oligomers into inert monomers, therein preventing the accumulation of oligomeric species.

Following the development of a highly reproducible aggregation assay and the quantification of the microscopic steps implicit to the Aβ42 aggregation reaction [69,70], numerous studies have been reported that dissected the specific molecular processes inhibited by candidate therapeutic compounds. Aβ42 aggregation is characterized by the formation of oligomers by primary nucleation, where a relatively low population grow and become fibrillar aggregates. This process is stochastically limited until a small but critical concentration of fibrillar aggregates have formed, after which time monomer-dependent secondary nucleation on fibril surfaces dominates the proliferation of further Aβ42 aggregates through a positive feedback loop [58,69,71]. During secondary nucleation, monomeric proteins are catalytically converted to oligomeric aggregates on the fibril surface, and these oligomers can then grow into fibrillar aggregates. The nature of this multiplicative process dictates that the majority of oligomers are formed from secondary pathways during the aggregation reaction once the critical concentration threshold of fibrillar aggregates is exceeded [72,73]. We note that these kinetic assays require highly pure monomeric peptide as the starting material, as the presence of higher-order aggregates at the start of an aggregation reaction modulates the mechanism of protein assembly [56,58,74]. Moreover, all assays based on amyloid-specific fluorescent dyes have the potential to generate false positive results, in particular when a prospective therapeutic shares structural similarity to the probe being used. For example, some conformations of candidate inhibitors, including quinones of catecholamines, polyphenols, and flavonoids, have been found to quench the fluorescence of thioflavin T (ThT), an amyloid-specific dye commonly used in aggregation assays [75]. It is therefore important to run independent validation experiments to confirm the mechanism of action of a candidate therapeutic, which can be accomplished through the use of label-free methods, including atomic force microscopy (AFM) [66,67,76,77], transmission electron microscopy (TEM) [76], or dot-blot assays [66] on aggregation mixtures over time using conformation or sequence-specific antibodies in the absence and presence of centrifugation. In doing so, it is possible to gain mechanistic insight into the microscopic steps governing the protein aggregation reaction using ThT kinetics while safeguarding against the possibility of false positives that can result from molecules that bind to amyloid aggregates and induce the displacement or quenching of ThT or other amylophilic dyes rather than directly impacting amyloid fibril formation [74].

By adopting this type of approach, the anticancer drug bexarotene was characterized as a potent inhibitor of primary nucleation in the Aβ42 aggregation process [56], and the subsequent derivatization of this small molecule found that structurally similar agents could inhibit different and specific microscopic steps, to include primary and secondary nucleation, to varying degrees [58]. A related strategy leveraged the high specificity of rationally designed antibodies to generate a library scanning the sequence of monomeric Aβ42, and a subsequent kinetic analysis revealed that the antibodies selectively targeted specific microscopic steps in a similar fashion [62]. Additionally, phage display was used to optimize the antibody inhibitory power by targeting specifically fibril-dependent secondary nucleation [78].

A recent study has compared the mechanism of action of four clinical stage antibodies (aducanumab, bapinezumab, gantenerumab, and solanezumab), revealing that while they all modulate Aβ aggregation, they do so with different effects on Aβ oligomers [68]. Aducanumab was found to be particularly effective in binding Aβ fibrils, thereby blocking the production of Aβ oligomers by secondary nucleation [68], in addition to directing the fibrils themselves towards degradation. This antibody is currently under consideration by the FDA and, if approved, will be the first disease-modifying treatment for AD. By contrast, the other three antibodies did not show major effects on Aβ oligomer production. Gantenerumab and bapinezumab were found to bind both Aβ monomers and fibrils, and to inhibit mainly fibril elongation, and solanezumab was found to bind Aβ monomers, and to inhibit primary nucleation. Similarly to aducanumab, BAN2401, which is a humanized version of an antibody raised in mice immunized with Aβ42 protofibrils [79] and is in Phase 3 clinical trials, has the ability to potently inhibit Aβ protofibril accumulation in AD mice [80]. Of note, crenezumab has been reported to target different Aβ conformations, including oligomers, and to reduce oligomer levels in cerebrospinal fluid (CSF) [81,82].

Molecular chaperones have also been characterized to attenuate Aβ42 aggregation, including DNAJB6, which targets primary nucleation [83], the transport protein transthyretin, which inhibits both primary and secondary nucleation processes in Aβ aggregation [77]; a Brichos domain, which inhibits specifically the monomer-dependent secondary nucleation microscopic step [63]; and clusterin, which attenuates fibril elongation at low concentrations [71]. The latter process may not be therapeutically relevant, as inhibiting elongation can redirect monomeric proteins from fibril ends to their surface, where autocatalysis leads to the generation of additional oligomeric and fibrillar aggregates. By quantifying a structure–kinetic–activity relationship for drug discovery against Aβ42, it was found that hit compounds, including those with minimal inhibitory power in their original form, could be rationally optimized from a kinetic perspective to target the production of oligomers [84]. Collectively, these results facilitate hope that hit compounds, including small molecules and antibodies, can be further optimized using rationally designed processes to target specifically the oligomeric toxins that are implicit and central to protein aggregation [58,62,84].

It has also been reported that naturally occurring polyphenols, including epigallocatechin-3-gallate (EGCG), curcumin, resveratrol, gallic acid, and oleuropein, as well as naturally occurring polyphenolic biflavonoids, and polyphenol molecules containing two flavone groups, such as amentoflavone, bilobetin, sequoiaflavone, sotetsuflavone, podocarpuflavone, ginkgetin, isoginkgetin, and sciadopitysin, can reduce the cytotoxicity of oligomers by inhibiting protein aggregation [85,86]. Many studies have shown that both polyphenols and polyphenolic bioflavonoids interact with the aggregation profiles of Aβ and αS either by destabilizing existing β-sheet rich fibrils to create less toxic monomers or through directly inhibiting the formation of soluble oligomers [85]. Studies have revealed that polyphenolic biflavonoids interact with the Aβ aggregation reaction through inhibiting Aβ fibrillization and disaggregating pre-formed fibrils, significantly reducing their cytotoxicity. Notably, bioflavonoids possess hydrophobic and hydrophilic groups that are proposed to facilitate their binding to toxic aggregates through hydrophobic and aromatic interactions, as well as hydrogen bonds. Additionally, biflavonoids have an increased number of aromatic rings compared to monoflavonoids, which may potentially afford them increased effectiveness in inhibiting fibrillization [85,86]. The interaction of curcumin, a natural polyphenol, with αS can induce both the disaggregation of large pre-formed aggregates, effectively creating more non-toxic monomers, as well as the prevention of oligomerization by increasing the solubility of αS [86,87].

Aminosterols, a family of molecules including trodusquemine and squalamine, can inhibit and accelerate protein aggregation depending on the protein under investigation [61,64,76]. Trodusquemine and squalamine inhibit αS oligomer formation [61,64], while trodusquemine has been found to accelerate Aβ aggregation [76]. Both aminosterols inhibit αS aggregation by preventing the lipid-induced initiation process [61,64], therein attenuating the rate of the oligomerization reaction. Further studies of trodusquemine revealed its ability to also inhibit fibril amplification in αS aggregation, demonstrating its ability to exert a multi-step inhibition mechanism [64].

Another class of molecules exhibiting anti-amyloidogenic behavior are tetracyclines and anthracyclines [86,88,89]. Tetracyclines, notably tetracycline and doxycycline, and the anthracycline iododoxorubicin, can disrupt pre-formed oligomers and reduce their toxicity [88,89]. Due to structural similarities between tetracyclines and anthracyclines, it has been proposed that the inhibition tendencies of these molecules originate from their binding to fibril structures. Moreover, a variety of probe and diagnostic dyes, including Congo red, methylene blue, crystal violet, acid fuchsin, and fast green FCF, have revealed the inhibitory capacity of these molecules [86,90,91,92,93].

Finally, recent studies have shown that it may be possible to target Aβ42 and αS in their monomeric forms to prevent their aggregation through the use of small molecules [66] or nanobodies [67]. From a kinetic perspective, monomer sequestration would be expected to reduce primary nucleation, secondary nucleation, and elongation [83], and despite the challenges associated with binding an intrinsically disorder protein, these approaches hold promise towards combatting the formation and proliferation of oligomeric species. Collectively, all of the inhibitors described in this section can function to suppress amyloid toxicity by reducing the number of toxins that are formed during the aberrant protein aggregation reactions central to numerous proteins and peptides.

### 3.2. Enhancing Protein Aggregation to Reduce the Populations of Oligomeric Species

Studies focused on preventing oligomer cytotoxicity through controlling the kinetics of their formation have revealed a limited set of molecules that act in a net beneficial way by accelerating the protein aggregation reaction. Molecules acting through the acceleration mechanism are postulated to function by shortening the lifetime of the oligomeric phase of the aggregation reaction by stimulating their conversion to the less toxic fibrillar form. Despite the possibility of the enhancement strategy as a potential mode to suppress oligomer toxicity, a relatively small number of studies have demonstrated the enhancement of fibril formation with the result that toxicity is reduced in comparison to the body of work that is established for inhibitory compounds.

Congo red has been reported to promote β-sheet formation and Aβ aggregation, which may act to reduce the lifetime and corresponding populations of oligomeric species, but it has also been suggested to attenuate aggregation by stabilizing monomeric or partially folded intermediates of the peptide [86,93]. Another dye, methylene blue, has demonstrated differential effects dependent upon the protein under investigation, where it was found to mitigate Aβ oligomerization through the promotion of Aβ fibrillization [92] and to inhibit prion protein aggregation [94]. The nonsteroidal anti-inflammatory drug sulindac sulfide similarly was characterized to deplete toxic Aβ oligomers by enhancing the rate of fibrillization in vitro [95], and the luminescent-conjugated oligothiophene p-FTAA was determined to suppress the number of toxic Aβ aggregates by generating amyloid fibrils that in this case were less hydrophobic and more resistant to proteinase K digestion [96].

Through a creative strategy, the racemic combination of mirror-image enantiomers of Aβ42 was shown to cause an acceleration in its rate of aggregation with a reduced propensity to form soluble oligomers, therein demonstrating that the enantiomeric mixing stimulated the formation of non-toxic fibrils [97]. The orcein-related small molecule O4 has also been characterized for its ability to bind hydrophobic amino acids in Aβ and catalyze the Aβ polymerization reaction. Therein, it was demonstrated that the O4-mediated acceleration of fibril formation decreases the concentration of oligomers in vitro and that O4 prevented oligomer-induced dysfunction in hippocampal brain slices, therein revealing that shifting the reactive flux of the aggregation reaction can suppress toxicity [98].

Recently, it was shown that the aminosterol trodusquemine, a natural product originally isolated from the dogfish shark, stimulates predominantly secondary nucleation in Aβ42 aggregation by solving analytically for the perturbation induced by the molecule using the master equation formalism [76]. This kinetic model was validated by measuring the morphology of the final fibrillar products formed in the absence and presence of trodusquemine using high-resolution and phase-controlled atomic force microscopy [99,100,101], which demonstrated that the aminosterol generated a multitude of shorter and wider fibrils that were consistent with the predicted molecule-induced shift in reactive flux [76]. As discussed in more detail in Section 5 of this review, aminosterols also possess the ability to displace toxic oligomers of multiple proteins from cell membranes [61,64,76,102]. In a *C. elegans* model of AD, we found that trodusquemine decreased the toxicity induced by Aβ42 aggregation alongside stimulating the rate of its aggregation [76], in stark contrast to its multistep mechanism of inhibition that was observed in vitro and in vivo with respect to αS aggregation [64]. These results for trodusquemine suggest that both the conversion of Aβ42 oligomeric aggregates to less toxic, higher-order fibrillar forms, and also their displacement from cell membranes can work in tandem with a combinatorial effect to suppress oligomer cytotoxicity [76,102].

### 3.3. Redirecting the Protein Aggregation Process to Sequester Oligomeric Species

In this section, we focus on potential therapeutics that can modulate the aggregation reaction in a way that redirects oligomeric aggregates towards less toxic, higher-order forms that are often off-pathway, e.g., high molecular weight aggregates, rather than mature fibrils. Despite the differing end species in these two processes, both off-pathway high molecular weight aggregates and fibrils can achieve the same result of reducing the lifetime or populations of oligomeric aggregates. Specifically, the formation of higher-order species by the redirection mechanism can consume toxic oligomeric intermediates, thereby depleting the populations of oligomers that are present and able to induce cellular dysfunction.

Resveratrol has the ability to redirect three separate conformers in the aggregation reaction to form unstructured off-pathway aggregates that are both non-toxic and of a high molecular weight [103]. These non-toxic aggregates can be formed by resveratrol from the soluble oligomers, fibrillar intermediates, and amyloid fibril conformers of the aggregation reaction, therein reducing toxicity by limiting the number of oligomers and other conformers that can participate in the aggregation reaction.

In addition to the polyphenol resveratrol, EGCG also reduces toxicity by remodeling mature amyloid fibrils and aggregates of Aβ and αS [104,105,106]. Studies show that EGCG remodels the large, mature Aβ and αS fibrils into unstructured and nontoxic aggregates, decreasing the amount of amyloidogenic species without creating a toxic product or reforming toxic oligomers in its mechanism of action [105]. A similar mechanism has also been displayed for other polyphenols in addition to resveratrol and EGCG, including trihydroxybenzophenone in tau protein aggregation and myricetin in Aβ aggregation [107,108].

The biological consequences of forming large deposits of aggregates in vivo as a result of redirecting the aggregation reaction away from fibrillar species, as well as the ability of the protein homeostasis machinery of the cell to store or degrade such high molecular weight aggregates, such as through the clearance of Aβ peptides through a proteasome-dependent mechanism, are processes likely to play an important role in determining the physiological relevance of this approach in patients afflicted by neurodegenerative disease. For example, fibrils have been shown to act as reservoirs of oligomers, thereby storing them for their ultimate release [37].

Collectively, there is a wide range of heterogeneity with respect to the function and specificity of molecular agents designed to disrupt the protein aggregation process with the result that toxicity is reduced through inhibiting, accelerating, or redirecting the aggregation of specific proteins. As can be observed by the appearance of certain molecules in the various classes of kinetic modulators of aggregation described herein, the Aβ, αS, and tau protein aggregation reactions have many steps by which molecules can interact with and perturb the direction and speed of the reaction in ways that can reduce oligomer toxicity. As described for small molecule inhibitors [58,84], rational strategies to optimize molecular potency could lead to species that can more efficiently accelerate or redirect the aggregation reaction with the potential to further reduce toxicity associated with the oligomeric state.

## 4. Targeting the Physicochemical Properties of Misfolded Protein Oligomers

Owing to the transient and metastable nature of oligomeric species formed during an aggregation reaction in vitro or in vivo, these aggregates represent a very small relative proportion of the total protein concentration. For example, it has been shown that oligomeric aggregates can reach a maximum of 1% of the total monomer concentration during an in vitro aggregation process for Aβ42 at relevant concentrations of the peptide [69,73]. In addition to their short lifetimes, oligomers of various proteins are highly heterogeneous in terms of their structures, morphologies, mechanisms of formation, and biological activates [109]. In light of these facts, a multitude of studies have used chemical or physical means to isolate or stabilize oligomeric aggregates in order to facilitate their investigation at pragmatic timescales and concentrations [45,50,110,111,112].

The exposure of hydrophobic patches and size are structural determinants of oligomer toxicity, and a high level of hydrophobicity and small size are associated with the ability to cause cellular dysfunction (Figure 1b) [31,37,57,113,114,115]. Multiple studies have established the contribution of increased solvent-exposed hydrophobicity to the toxicity of Aβ oligomers [57,116,117]. Additionally, the size of the oligomers themselves is a key factor, with larger oligomers being less toxic than their smaller counterparts [57,117,118]. Consequently, numerous studies have examined methods for reducing the solvent-exposed hydrophobicity and increasing the size of oligomers for therapeutic purposes. We therefore focus herein on the size–hydrophobicity–toxicity relationship.

By generating 12 oligomer variants of the N-terminal domain of the *E. coli* HypF protein (HypF-N), it was demonstrated that the induced variations in oligomer size and hydrophobicity rationalized the differential empirically observed cellular dysfunction caused by the aggregates. Moreover, an equation was proposed based on size and solvent-exposed hydrophobicity that could predict the toxicity of oligomers using relatively high-throughput methods [57]. This equation was recently leveraged to explain the effects of rationally designed antibodies that were used as molecular tools to probe the size–hydrophobicity–toxicity relationship for zinc-stabilized Aβ40 oligomers [50], where concomitant increases in size and hydrophobicity induced by the antibodies were predicted and observed not to change the toxicity of the stabilized oligomers due to their offsetting effects [117]. Moreover, two forms of αS oligomers have been resolved to embed themselves in cell membranes to different degrees based upon their levels of solvent-exposed hydrophobicity, therein disrupting membrane integrity only in the case of the embedded variant that was more significantly more hydrophobic [55].

The concept of reducing the cellular toxicity of oligomeric species by their sequestration into larger, innocuous species with reduced diffusional mobility has been accomplished previously through the use of molecular chaperones [113,114,119,120] and small molecules [103,106]. In particular, a variety of chaperone and non-chaperone proteins have been shown to suppress the toxicity of oligomers by promoting their assembly into bigger and less mobile species [114], including Hsp27 with Aβ42 oligomers [120] and other molecular chaperones with Aβ42, IAPP, and HypF-N oligomers [113]. In some cases, the mechanism of action of these molecular chaperones involves the inhibition of the interaction of oligomers with cellular membranes [121,122]. Given their propensity to target oligomeric intermediates, many of these chaperones also modulate the kinetics of protein polymerization, as discussed in the preceding sections. Indeed, multiple chaperones can play an important role in regulating the aggregation reactions innate to AD [63,123,124,125]. Molecular chaperones, such as clusterin and crystallin, also protect cells from the aggregation of misfolded proteins by modulating the folding of their respective substrate proteins [126].

Heat shock proteins are a class of molecular chaperones that have received attention for their ability to reduce Aβ42 cytotoxicity; HspB1 and Hsp70 have both been shown to modulate the cytotoxicity of Aβ42 in vitro [120,127], although they operate through different mechanisms. Of the two, HspB1 appears to function by modulating the size–hydrophobicity–cytotoxicity relationship, sequestering oligomers into relatively inert aggregates [120], whereas Hsp70 appears to disrupt the primary nucleation process of oligomerization [127] by interacting with the hydrophobic residues of Aβ42 [128]. Since heat shock proteins are found to be co-located with amyloid plaques in the brains of AD patients, it is possible that increasing their endogenous concentration in vivo could improve outcomes for patients. In support of this notion, transgenic mouse models for AD that are deficient in heat shock proteins express significantly worse symptoms than their counterparts who do have the ability to produce heat shock proteins [129], suggesting that these chaperones play an important role in regulating Aβ42 aggregation.

In addition to heat shock proteins, a number of other species have shown promise in modulating the size or hydrophobicity of Aβ42 aggregates. For example, monomeric human transthyretin has been shown to produce large co-aggregates with reduced cytotoxicity and inhibit nucleation processes [130]. Clusterin can also bind to hydrophobic patches in Aβ42 oligomers and inhibit its aggregation [131]. The aminosterol trodusquemine accelerates the aggregation of Aβ42 in vitro, reducing the concentration of toxic oligomers in solution and encouraging the formation of fibrillar aggregates [76]. Analogous to the case of the rationally designed antibodies against oligomers of Aβ40 stabilized by zinc ions, trodusquemine at super-stoichiometric concentrations that are not physiologically relevant was observed to linearly increase the size and hydrophobicity of the stabilized aggregates. These physiochemical chemical changes would therefore be predicted not to change the toxicity of the oligomers [117], and it was shown that these biophysical parameters were not overtly changed at physiological concentrations [102]. Collectively, these studies support the existence of a size–hydrophobicity–toxicity relationship and suggest that targeting these biophysical parameters can result in alleviating the cytotoxicity innate to protein misfolded oligomers by reducing oligomer binding to cell membranes or specific receptors.

## 5. Targeting the Aberrant Interactions of Misfolded Protein Oligomers

A relatively distinct mechanism of counteracting the cytotoxicity of misfolded protein oligomers is through the use of molecules to prevent their deleterious interactions with cell membranes. Oligomeric species of Aβ42 are thought to damage cells by binding to their membranes, therein forming ion channels and disrupting the integrity of the cell membrane [132,133]. Recent work suggests that cellular membranes can act as nucleation sites for Aβ42 oligomerization at physiological concentrations of the protein [134]. Cell membranes themselves are highly heterogenous in their compositions of various biomolecules, including sterols, fatty acids, lipids, and scaffolding proteins, amongst many other molecules, and it has been suggested that age-related changes in membrane lipid rafts correlate to the loss of neuronal function observed in neurodegenerative disease [135]. Furthermore, Aβ42 oligomers bind to a wide variety of receptors on membranes, which has contributed significantly towards the challenge of blocking the effects of oligomer binding to membranes by targeting a specific receptor or set of receptors [136].

The environment of the cellular membrane, with its heterogeneous regions of differing lipids and receptors, provides evidence for the proposed general mechanism of oligomeric membrane interactions. For one, extensive research has demonstrated that the ganglioside GM1 (and ganglioside GT1b to a lesser degree) amplifies Aβ42 cytotoxicity by promoting its aggregation cascade [137]. Additionally, evidence suggests lipid rafts as regions of increased oligomer interaction with the membrane, as well as the site of the cleavage of amyloid precursor protein (APP) [138]. Furthermore, studies have identified relatively expanded regions of the membrane characterized by increased cholesterol content as more favorable for the insertion of APP [139], which may be related to the increased distribution of cholesterol on the exterior of lipid rafts as cells age [138]. Moreover, the sphingolipid sphingomyelin is thought to be a driver of lipid raft formation due to its interaction with cholesterol, pointing to its potential influence on Aβ42–membrane interactions [140]. The aggregation of Aβ42 activates protein homeostasis systems in the cell, which downregulates sphingomyelin production, further implicating sphingomyelin and by extension lipid rafts in the progression of AD [141]. Finally, an increase in sphingomyelin synthesis is associated with oxidative stress and increased concentrations of long-chain ceramides, which can induce cellular stress responses [142]. These interactions, when taken together, indicate that while oligomer binding to specific membrane receptors does indeed occur, the mechanism of membrane interaction appears to be more generic in nature.

While the structures of Aβ42 oligomers are currently not well resolved, with many different shapes and structures having been characterized simply in the case of Aβ oligomers, such as dimers [143,144,145], timers [146,147], Aβ*56 oligomers [32,148], ADDLs [25,26,149], and amylospheroids as spherical Aβ oligomers [150], where each type of oligomer can exhibit differing biological effects [151], a common principle exists: toxic oligomeric species bind to cellular membranes and disrupt membrane integrity [37]. Due to their high degree of solvent-exposed hydrophobicity, oligomers embed into the interior of the membrane, remaining relatively immobile with respect to the rest of the membrane, with a majority of oligomers remaining in place rather than diffusing throughout the membrane [152,153]. The oligomers, once embedded, disrupt the function of the cell by increasing the molar volume of acyl chains in the interior of the membrane, thereby increasing the conductivity of the membrane, most notably to calcium ions [53,154]. Additionally, the method of binding of oligomers to cell membranes has been observed to be heterogeneous in nature, with the same peptide binding to different regions of the membrane based on its specific quaternary structure, which further points toward a general mechanism by which oligomers interact with membranes, rather than one beholden to a specific lipid moiety [152,153]. Therefore, molecules that displace misfolded protein oligomers from the cell membrane stand to potentially disrupt both aggregation pathways and directly protect cells from the effects of already assembled oligomers [61,155].

One promising molecule that operates according to this mechanism is again the aminosterol trodusquemine, which can cross the blood–brain barrier [156]. Trodusquemine displaces a variety of toxic oligomers from cell membranes in vitro, including those of Aβ40, Aβ42, HypF-N, and αS, suggesting that the aminosterol-induced protection of cell membranes from oligomers occurs through a generic mechanism (Figure 1c) [64,76,102]. This oligomer displacement hypothesis for the mechanism by which trodusquemine attenuates oligomeric cytotoxicity is also supported by research observing a marked difference in the impact of trodusquemine on the aggregation of Aβ42 and αS, where the former was enhanced and the latter inhibited, coupled with similar reductions in the cytotoxicity of isolated or stabilized oligomers [64,76,102]. These studies together suggest that trodusquemine is able to outcompete oligomers for binding sites on cell membranes. Importantly, this mechanism does not seem to effect the normal binding of monomeric αS to cell membranes [64].

Similar to trodusquemine, other aminosterols have been shown to function through a comparable mechanism. Specifically, squalamine reduces oligomer toxicity in a similar fashion [61]. In addition to the aminosterol family, cholesterol has also been shown to impact the binding of Aβ40 oligomers to cellular membranes at super-stoichiometric concentrations [157], and a clear correlation was found between the concentration of GM1 and binding and subsequent toxicity of Aβ42 and HypF-N oligomers [158]. Additionally, the small molecule anle138b appears to affect Aβ42 oligomers in the cell membrane, binding to the pores Aβ42 creates in membranes and thereby preventing the loss of membrane integrity normally associated with Aβ42 membrane interactions [159].

Collectively, these studies illustrate the importance of membrane composition with a wide range of biomolecules and show that molecules that interact directly with cell membranes, rather than binding oligomeric aggregate themselves, represent an approach distinct from the previous strategies. By bypassing the heterogeneity innate to protein oligomerization and the host of specific receptors that may play a role in the oligomer-mediated dysfunction of cell membranes via targeting membranes in a ubiquitous manner, molecules with a common therapeutic effect on a variety of toxic oligomers have been identified [102]. Further exploration of molecules that compose and interact with membranes, therefore, could be a promising avenue by which to arrive at cellular targets for oligomeric species and potential countermeasures against their deleterious effects.

## 6. Targeting Misfolded Protein Oligomers by Potentiating the Protein Homeostasis System

**Figure 2:**
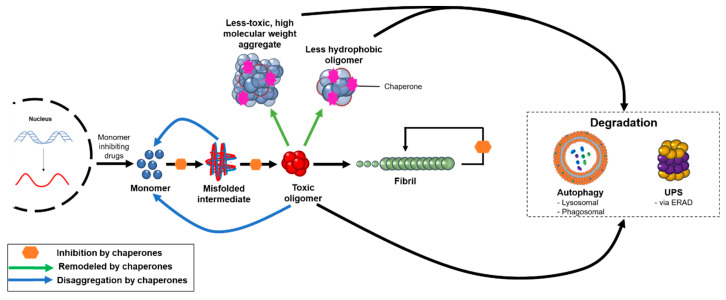
The protein homeostasis system targets oligomeric aggregates through a variety of mechanisms. Molecular chaperones can redirect misfolded or aggregated proteins back to the monomeric state, remodel pre-formed oligomers into less hydrophobic or higher molecular weight aggregates that are less toxic, and inhibit various microscopic steps in the protein aggregation process. These aggregates can be targeted for degradation by autophagic and proteasomal processes, and the degree to which the body can eliminate protein aggregates plays a critical role in the onset and progression of neurodegenerative disease. In addition, therapeutics are under development to reduce the production of monomeric proteins, such as through the use of BACE1 inhibitors in AD.

In order to be functional, the proteins that constitute the cellular proteome need to fold properly and remain soluble. The maintenance of a functional proteome is achieved through the action of the protein homeostasis system, which regulates the synthesis, folding, transport, and degradation of proteins, guaranteeing their functionality and mitigating their aggregation [160]. This system assists in protein folding, remodeling misfolded proteins back to folding intermediates, disaggregating intermediate aggregates, and assisting in protein turnover or aggregate removal via the ubiquitin–proteasome or autophagosomal–lysosomal systems (Figure 2) [51,161]. This quality control system declines over time as a function of ageing, often resulting in age-related comorbidities [161,162]. Over the course of a life, the gradual accumulation of certain mutations can result in a larger pool of aggregation-prone species. Evidence from model organisms suggests that as genetic and epigenetic longevity controls affect a downregulation in the expression of molecular chaperones, it becomes increasingly difficult for the protein homeostasis network to adequately keep pace with the growing demand of proteotoxic species [51].

The decline of the protein homeostasis network represents both a trigger and a target in a wide range of protein misfolding diseases. A study in *C. elegans* showed that naturally the vast majority of the proteins are expressed at concentrations close to their intrinsic solubility limits [15], and these supersaturated proteins form a metastable subproteome that is highly susceptible to aggregation related to specific neurodegenerative diseases [13,16].

Primary regulators of the protein homeostasis system are molecular chaperones, which constitute a family of structurally and functionally diverse proteins with varying mechanisms of action based on cell-specific expression [163]. According to a recent mapping, the human chaperome consists of 332 genes, of which 88 are molecular chaperones and 244 are co-chaperones [164]. As humans age, the quality of these control measures often degrades leading to the formation and propagation of toxic protein aggregates, specifically intermediate oligomers that are commonly found in neurodegenerative diseases. While the role that molecular chaperones play in maintaining protein homeostasis is well documented [165,166], recent studies have shown that they also exhibit cytoprotective characteristics through their ability to neutralize toxicity associated with aberrant protein oligomers present in neurodegenerative diseases [114,164]. Numerous studies have shown the ability of molecular chaperones to interact with oligomers, and fibrils, to neutralize their associated toxicity by preventing cell membrane interaction and inducing the formation of larger, non-toxic species [114,167,168], and others have demonstrated the ability to inhibit protein aggregation and therefore oligomer formation [63,127]. While many mechanisms of action of molecular chaperones are not fully resolved, they may be utilized therapeutically to reduce the deleterious effects of oligomers in protein misfolding diseases. Recently, the S100 family of proteins have been investigated for their role in AD, based on their ability to influence the Aβ aggregation pathway and its cellular receptors. While evidence shows that their function depends on the stage of pathology and related levels of Aβ and S100 proteins, it is possible that the S100 family could function as molecular chaperones to suppress oligomer toxicity through several mechanisms [169,170]. Development of chaperone-targeted therapeutics is limited by their stability, oral bioavailability, and blood–brain barrier permeability. Current therapeutic strategies targeting molecular chaperones include development of small molecule inhibitors or activators, viral and non-viral mediated delivery of molecular chaperones or their activators, and pharmacological chaperones [171,172,173,174,175,176].

This type of approach may be particularly helpful in the case of neurodegenerative diseases such as PD, which have been linked to the dysfunction of lipid metabolism resulting in neuroinflammation [177]. The involvement of the stress-induced unfolded protein response (UPR) of the endoplasmic reticulum (ER) is an active area of investigation, and molecular chaperones are being considered to potentiate the folding capacity of proteins in the endoplasmic reticulum or mitochondria [161]. Targeting such stress responses by modulating signaling pathways could be therapeutically efficacious against the widespread toxicity caused by protein aggregation, as stress responses can vary significantly amongst different cell types.

A major therapeutic approach towards reducing oligomer numbers is to stimulate their removal from the cellular milieu. A promising target is autophagy, a lysosomal degradation pathway already investigated in relation to diverse pathologies including cancer and infections [178]. The pathway itself also deteriorates with age, which contributes to the myriad of comorbidities in elderly populations [179,180], and stimulation of the pathways that preserve autophagy may demonstrate a protective effect against protein aggregation [181]. For example, rapamycin has been demonstrated to inhibit mTOR signaling and to reduce the amount of amyloid plaques and tau tangles in an AD mouse model [182,183]. Contrasting studies, however, have suggested that inducing autophagy could cause deleterious changes in Aβ production, and it is currently unclear if alterations in autophagy are causative, protective, or simply a result of AD [184]. In addition to the autophagosomal–lysosomal pathway, aggregate removal can also be potentiated by the ubiquitin–proteasome system (UPS) [185]. This pathway requires ATP usage to unfold proteins, and in both systems, molecular chaperones recognize misfolded proteins and hold them in conformations that can be degraded [161,186].

In addition to enhancing the clearance of oligomer aggregates, reducing the concentration of Aβ through the use of secretase inhibitors or regulating its metabolism by ApoE-modifying therapeutics are active areas of clinical research [187,188]. Apolipoprotein E4 (ApoE4) is one of the three major isoforms of apolipoprotein E (ApoE) implicated in neurodegenerative disorders, in particular AD. These lipoproteins play an essential role in lipid metabolism, as well as maintaining normal brain function [189]. Studies have shown a strong association between ApoE, especially ApoE4, and Aβ accumulation in the brain leading to AD through reducing microglial function, lipid transport, synapse integrity and stability, energy metabolism, protein aggregation, and inhibition of protein clearance [190]. In these cases, reducing the populations or lifetimes of oligomers by acting on protein expression or degradation could prove therapeutically beneficial by indirectly targeting oligomeric species.

## 7. Discussion

The development of therapeutics for the prevention or treatment of protein misfolding diseases is fraught with challenges. For one, as misfolded protein oligomers are intermediates in the aggregation process, their structures are transient and heterogeneous. Furthermore, there are many interconnected pathways in the aggregation process in each disease, making the exact nature of the cytotoxic aggregates difficult to discern. For similar reasons, the oligomers themselves are also difficult to control in vitro, making estimates of oligomer concentrations and cytotoxic properties difficult to compare across different experimental protocols. By increasing our understanding of the mechanisms of action of candidate compounds targeting oligomers, we anticipate that it will become possible to develop more clinically relevant therapeutics in comparison to the numerous ones described thus far that target non-specifically the protein aggregation reaction and have failed in the clinical setting [3].

The molecules with the different mechanisms of actions outlined in this review (Figure 1 and Figure 2) have advantages and disadvantages. We have highlighted numerous candidate compounds for their ability to target amyloid oligomers in order to illustrate the current state of the field. To broaden the scope of this review and to show more examples of amyloid oligomer targeting species that fit within these categorizations, Table 1 summarizes compounds discussed in this review, as well as many more not mentioned explicitly in the text, for their ability to impact oligomers and reduce their toxicity in vitro, in model organisms, and, in some cases, in clinical trials. For the compounds in Table 1, the National Institutes of Health (NIH) U.S. National Library of Medicine’s clinical trial database (ClinicalTrials.gov) was used to determine the statuses of the various listed molecules that have been, or are currently in, clinical trials.

Modulating the kinetics of oligomer assembly by either acceleration, inhibition, or redirection has the advantage of working directly with the protein in question, and an extensive list of promising molecules have already been identified for their ability to suppress the toxicity innate to amyloid aggregation. Molecules that accelerate the aggregation cascade or redirect it toward the formation of larger, less cytotoxic aggregates, however, could lead to the long-term stabilization of sizable aggregates in the brain, which could cause complications over time.

Molecular chaperones, including those that modulate the size–hydrophobicity–toxicity relationship, have been intensively studied, and many promising candidates have been identified, including those that are dysfunctional in AD patients. However, these molecules are relatively large and typically unable to cross the blood–brain barrier, thus hindering their usefulness as therapeutics. Additionally, there is the question of whether these molecules, and other therapeutics that target the protein directly, will remain effective at relatively high concentrations of the protein, as most patients with neurodegenerative diseases are first diagnosed at an advanced stage of the disease.

Molecules that interact with oligomers on cell membranes have the advantage of functioning outside the aggregation cascade altogether, therein protecting cells in a general way, independent of the stage of oligomerization. Additionally, some of these molecules function through a generic mechanism and are therefore able to displace multiple oligomeric species, indicating their potential for use in treating multiple diseases [102]. However, these molecules also face challenges due to their mechanism of interaction, as they have varied effects on the aggregation cascades of different proteins related to neurodegenerative disease [61,64,76]. Additionally, since many of these molecules, such as trodusquemine, function by competing with oligomers at the site of membrane binding, their effectiveness would rely on their continual presence of the molecule for duration of the life of a patient.

Finally, modulation of the protein homeostasis network has proven therapeutically challenging, and strategies to reduce monomeric concentrations of disordered proteins have in some cases resulted in worsened cognition in AD patients, such as those for secretase inhibitors [187], highlighting the challenges of regulating the functional forms of the target proteins. For all described strategies, specificity for oligomeric aggregates remains a critical challenge in the design of effective therapeutics [86].

Looking forward, these classifications of potential therapeutic molecules illustrate that it may be possible to leverage combination therapies to combat neurodegeneration, as seen in many cancer treatments. Combining two molecules with different mechanisms of action has the potential to operate along the advantages of both methods, without engaging in competition.

Although not discussed in depth in this review, the treatment of neurodegenerative disease has proven highly challenging in significant part due to limited diagnostic assays that can detect the various pathologies early in a patient’s life. Recent efforts to rationally design antibodies against oligomeric Aβ42 have illustrated the possibility of using these highly specific molecular tools to advance diagnostics [191]. Moreover, advances in cryo-electron microscopy have recently led to the characterization of patient-derived fibrils of the tau protein in AD [192], Pick’s disease [193], chronic traumatic encephalopathy [194], corticobasal degeneration [195,196], and αS filaments from multiple system atrophy [197]. For the various tau fibrils in the above pathologies, discrete core structures were observed in a disease-specific manner. With recent advances in the resolution of this approach [198], and the possibility of isolating a homogenous population of oligomers from brain tissue, cryo-electron microscopy may in the future lead to a better understanding of the precise molecular nature of oligomers implicit in specific neurodegenerative diseases.

In conclusion, the efficacy of the different approaches described here will become clearer as the field advances to understand and combat the toxic effects of oligomers in neurodegenerative diseases.

## Figures and Tables

**Table 1:** Examples of compounds, including those that are currently in clinical trials, reported to target misfolded protein oligomers.

Compound	Molecular Family	Target	Proposed Mechanism of Targeting Oligomers
ABBV-0805 (BAN0805)	Antibody	αS aggregates, in particular oligomers and protofibrils	Humanized form of the murine antibody mAb47 that inhibits the accumulation of αS aggregates within astrocytes [199]
Aducanumab	Antibody	Aβ aggregates, including oligomers and fibrils	Autoantibody-derived antibody that binds Aβ oligomers and fibrils and reduces insoluble amyloid plaques [68,200]; under FDA review for approval
BAN2401	Antibody	Aβ aggregates, in particular protofibrils	Binds Aβ protofibrils, its murine version (mAb158) reduces levels of protofibrils in the brain and CSF of AD mice [80] and prevents Aβ accumulation in astrocytes [201]; in a Phase 3 clinical trial
Bapineuzumab	Antibody	Aβ, in particular soluble Aβ and fibrils	Passive Aβ immunotherapy; failed in a Phase 3 trial for AD despite biomarker changes in APOE ε4 carriers [68,202]
Cinpanemab (BIIB054)	Antibody	αS aggregates	Autoantibody-derived antibody that binds aggregated forms of αS and prevents its spreading; in a Phase 2 clinical trial [203]
Crenezumab	Antibody	Aβ aggregates, including oligomers and fibrils	Targets Aβ oligomers, fibrils, and plaques and reduces their levels in the CSF [81,82]; terminated in a Phase 3 clinical trial
Gantenerumab	Antibody	Aβ aggregates, in particular oligomers	Targets Aβ oligomers and reduces amyloid plaques [68,204]; in a Phase 3 clinical trial
Lu-AF-82422	Antibody	αS aggregates	Prevents the cell-to-cell transmission of αS [205]; in a Phase 1 clinical trial
MEDI1341	Antibody	αS aggregates, from monomers to higher-order species	Binds soluble and insoluble aggregate forms of αS [206]; in a Phase 1 clinical trial
PMN310	Antibody	Aβ aggregates, in particular oligomers	Inhibits Aβ oligomer propagation and toxicity [207]
Prasinezumab (PRX002)	Antibody	αS aggregates	Reduces truncated forms and slows αS propagation [208]; in a Phase 2 clinical trial
Rationally designed antibodies	Antibody	Aβ aggregates, in particular oligomers and fibrils	Selectively inhibits specific microscopic steps in Aβ42 aggregation [62] or binds specifically Aβ oligomers [191]
Solanezumab	Antibody	Aβ aggregates, in particular soluble forms	Binds soluble forms of Aβ and promotes its clearance from the brain in pre-clinical models [68,209]; did not meet endpoints in two Phase 3 trails, with one Phase 3 trial currently ongoing
Sargramostim (Leukine)	Glycoprotein	Granulocyte-macrophage colony-stimulating factor	Stimulates the innate immune system to suppress Aβ oligomer levels in mice [65,210]; Phase 2 clinical trial completed
Docosahexaenoic acid	Lipid (fatty acid)	α-, β-, and γ-secretases	Reduces amyloid production by decreasing β- and γ-secretase activity and increases nonamyloidogenic processing by stabilizing α-secretase [211]
Monosialotetrahexosylganglio-side GM1	Lipid (glycosphingolipid)	Cell membranes	Increases oligomer binding and subsequent toxicity for Aβ42 and HypF-N oligomers [158]
Cholesterol	Lipid (sterol)	Aβ aggregates and cell membranes	Can affect the binding of Aβ oligomers to cell membranes [157], and catalyzes the heterogeneous nucleation of Aβ42 [212]
Brichos domain	Molecular chaperone	Protein aggregates, in particular fibrils	Inhibits monomer-dependent secondary nucleation in Aβ42 aggregation [63]
Clusterin	Molecular chaperone	Protein aggregates, in particular fibrils	Attenuates Aβ fibril elongation at low concentrations [71]
Crystallin	Molecular chaperone	Protein aggregates	Can protect cells from protein aggregation by modulating protein folding [126]
DNAJB6	Molecular chaperone	Protein aggregates, in particular oligomers	Targets and inhibits primary nucleation in Aβ42 aggregation [83]
Hsp27	Molecular chaperone	Protein aggregates, in particular oligomers	Suppresses oligomers toxicity by promoting their assembly into larger, innocuous species with reduced diffusional mobility [114] for Aβ42 oligomers [120]
Hsp70	Molecular chaperone	Protein aggregates	Can disrupt primary nucleation processes of oligomerization [127] by bonding to the hydrophobic regions of Aβ42 [128]
HspB1	Molecular chaperone	Protein aggregates, in particular oligomers	Modulates the size–hydrophobicity relationship, sequestering oligomers into relatively inert aggregates [120]
100074-G5	Small molecule	Monomeric Aβ42	Binds the monomeric state of Aβ42 and prevents primary and secondary nucleation processes [66,213]
ALZ-801	Small molecule	Aβ aggregates	Inhibits Aβ oligomer formation [204]; Phase 1 clinical trial completed
ANAVEX2-73 (Blarcamesine)	Small molecule	Aβ aggregates	Reversed learning deficits in mice injected with Aβ25-35 and prevented hippocampal oxidative stress [214]; Phase 2 clinical trial completed
anle138b	Small molecule	Cell membranes and protein aggregates	Blocks the activity of conducting Aβ pores in cell membranes [159]; Binds αS aggregates and prevents loss of membrane integrity associated with αS membrane interactions [215]; Phase 1 clinical trial completed
Candesartan cilexetil	Small molecule	Aβ aggregates, in particular oligomers	Prevents Aβ40 and Aβ42 oligomerization in vitro [216]; Phase 2 clinical trial completed
Cilostazol	Small molecule	Aβ aggregates, in particular oligomers	Reduces Aβ oligomerization and toxicity, and promotes Aβ clearance [217,218]; clinical trials ongoing
CNP520 (Umibecestat)	Small molecule	BACE1 inhibitor	Reduces brain and CSF Aβ levels in rats, dogs, AD mice, and humans [219]; in a Phase 2/3 clinical trial
Congo red	Small molecule	Protein aggregates	Disaggregates oligomers by solubilizing them, promotes β-sheet formation [93], attenuates aggregation by stabilizing monomeric or partially folded intermediates of the peptide [86,93]
Cromolyn sodium (as part of ALZT-OP1)	Small molecule	Aβ aggregation, in particular monomers and oligomers	Inhibits Aβ aggregation and promotes its clearance [220]; ALZT-OP1 is in a Phase 3 clinical trial
Crystal violet, acid fuchsin, fast green FCF, symmetrical cyanide inhibitors	Small molecule	Protein aggregates	Inhibit tau aggregation [86,221]
Curcumin	Small molecule	Protein aggregates, from monomers to fibrils	Inhibits Aβ and tau oligomerization, disrupts mature Aβ, tau, and αS fibrils, redirects Aβ and αS aggregation reactions to create nontoxic oligomers [85,86]; various past and ongoing clinical trials
Doxycycline, tetracycline	Small molecule	Aβ aggregates, in particular oligomers	Disrupts pre-formed Aβ oligomers and fibrils and inhibits fibril formation [88,89]
Elenbecestat (E2609)	Small molecule	BACE1 inhibitor	Reduces the concentration of Aβ by inhibiting its production [222]; in a Phase 3 clinical trial
Elyata (CT1812)	Small molecule	Oligomer receptors	Allosterically binds the sigma-2-receptor to displace Aβ-oligomers and reduce their toxicity [223,224,225]; in a Phase 2 clinical trial
Epigallocatechin-3-gallate (EGCG)	Small molecule	Protein aggregates from monomers to fibrils	Binds to monomers and prevents aggregation, remodels mature amyloid fibrils and aggregates of Aβ and αS into larger, nontoxic aggregates, [85,86,104,105,106]; many past and ongoing clinical trials
Furosemide	Small molecule	Aβ aggregates, in particular oligomers	Prevents Aβ40 and Aβ42 oligomerization and decreases Aβ oligomers levels in Tg2576 mice [216]
Gallic acid	Small molecule	αS aggregates, in particular soluble species	Inhibits αS amyloid fibril formation [85,86,226]
ID1201	Small molecule	α-secretase	Reduces Aβ and amyloid levels in AD-model mice by activating the PI3K/Akt pathway [65,227,228]; Phase 2 clinical trial completed
Iododoxorubicin	Small molecule	Protein aggregates, in particular fibrils	Disrupts fibril formation [88,89]; Phase 2 clinical trial completed
Methylene blue	Small molecule	Protein aggregates, in particular fibrils	Promotes Aβ fibrillization to deplete oligomers [92], inhibits tau [229] and prion protein aggregation [90,91,92,93,94]; TRx0237 is in a Phase 3 clinical trial
Nilotinib	Small molecule	Antineoplastic tyrosine kinase inhibitor	Reduces αS accumulation and tau hyperphosphorylation; in a Phase 2 clinical trial [230,231]
NPT200–11	Small molecule	αS aggregates, in particular oligomers	A small molecule rationally designed to target αS oligomers [232]
O4	Small molecule	Aβ aggregates	Binds to hydrophobic amino acids in Aβ and catalyzes the Aβ polymerization reaction to deplete oligomer populations [98]
Oleuropein	Small molecule	Protein aggregates	Prevents oligomer formation, disrupts oligomer binding to the plasma membrane and inhibits their toxicity [85,86]; associated with a dementia management clinical trial
Oligothiophene p-FTAA	Small molecule	Protein aggregates	Suppresses Aβ aggregation by generating amyloid fibrils that are less hydrophobic and resistant to proteinase K digestion [96]
PBT434	Small molecule	αS aggregates	Inhibits αS aggregation by preventing αS interactions with iron [233]
Posiphen	Small molecule	APP inhibitor	Targets APP mRNA to reduce APP and Aβ levels [234,235], also targets SNCA MRNA to reduce αS expression [236,237]; in Phase 1 (AD) and Phase 2 (PD) clinical trials
PTI-125	Small molecule	Restores native forms of filamin A, reduces associations with α7-nAChR/TLR4	Reduces tau hyperphosphorylation, Aβ42 deposition, neurofibrillary tangle formation, and neuroinflammation in 3xTg-AD mice [238]; in a Phase 2 clinical trial
Rapamycin	Small molecule	Autophagy stimulant	Inhibits mTOR signaling and reduces the amount of amyloid plaques and tau tangles in AD model mice [182,183]; in a Phase 1 clinical trial
Resveratrol	Small molecule	Protein aggregates	Redirects conformers in the aggregation reaction to form less toxic aggregates [85,86,103]; Phase 2 clinical trial completed
Squalamine	Small molecule	Protein aggregates, in particular oligomers and fibrils, and cell membranes	Displaces αS oligomers from cell membranes and inhibits αS lipid-induced nucleation [61]; ENT-01 (an aminosterol) is in a Phase 2 clinical trial (PD)
Sulindac sulfide	Small molecule	Aβ aggregates	Depletes toxic Aβ oligomers by enhancing the rate of fibrillization in vitro [95]
Trodusquemine	Small molecule	Protein aggregates, in particular oligomers and fibrils, and cell membranes	Displaces Aβ40, Aβ42, αS, HypF-N oligomers from cell membranes [64,76,102], inhibits αS lipid-induced nucleation and fibril amplification [64], enhances Aβ42 aggregation [76]; ENT-01 (an aminosterol) is in a Phase 2 clinical trial (PD)
Verubecestat (MK-8931)	Small molecule	BACE1 inhibitor	Reduces plasma, CSF, and brain levels of Aβ in rats and monkeys [239]; Phase 1 clinical trial completed
Bexarotene and derivatives	Small molecules	Aβ aggregates	Inhibit specific microscopic steps in Aβ42 aggregation [56,58]
Amentoflavone, bilobetin, sequoiaflavone, sotetsuflavone, podocarpuflavone, ginkgetin, isoginkgetin, sciadopitysin	Small molecules (bioflavones)	Aβ aggregates, in particular fibrils	Inhibits Aβ42 fibrillization and disaggregates pre-formed fibrils [85,86]
Trihydroxybenzophenone, myricetin, tannic acid	Small molecules (polyphenols)	Protein aggregates	Inhibit Aβ aggregation [240,241] and remodels tau fibrils into unstructured and nontoxic aggregates [107,108]
Human umbilical cord mesenchymal stem cells	Stem cell therapy	Soluble amyloid	Secretes soluble intracellular adhesion molecule-1 that decreases Aβ levels by inducing expression of the Aβ-degrading enzyme neprilysin [242]; in a Phase 1 clinical trial
Monomeric human transthyretin	Transport protein	Protein aggregates	Inhibits both primary and secondary nucleation in Aβ aggregation [77]
AADvac1	Vaccine	Tau aggregates, in particular phosphorylated tau	Reduces tau hyperphosphorylation and prevents its oligomerization [243]; in a Phase 1 clinical trial
ABBV-8E12	Vaccine	Protein aggregates, in particular tau	Removes brain and plasma tau and reduces tau pathology and associated atrophy [244]; in a Phase 2 clinical trial
CAD106	Vaccine	Aβ aggregates, in particular monomers and oligomers	Blocks Aβ toxicity in cell cultures, reduces amyloid accumulation in AD model mice, induces an immunogenic response [245]; in a Phase 2 clinical trial
UB-311	Vaccine	Aβ aggregates, from monomers to higher-order aggregates	Preferentially binds to higher-order Aβ42 aggregates and reduces Aβ42 oligomer, protofibril, and plague levels by stimulating an immunogenic response [246]; in a Phase 2 clinical trial

## Abbreviations

αS	α-synuclein
Aβ	amyloid-β peptide
Aβ40	40-residue form of the amyloid-β peptide
Aβ42	42-residue form of the amyloid-β peptide
ADDLs	Aβ-derived diffusible ligands
AFM	atomic force microscopy
ApoE	Apolipoprotein E
APP CSF	amyloid precursor protein cerebrospinal fluid
EGCG	epigallocatechin-3-gallate
ER	endoplasmic reticulum
GM1	monosialotetrahexosylganglioside
HypF-N	N-terminal fragment of the HypF protein
IDP	intrinsically disordered protein
*k*_1_	primary nucleation (microscopic step)
*k*_2_	secondary nucleation (microscopic step)
*k*_+_	elongation (microscopic step)
PD	Parkinson’s disease
UPR	unfolded protein response
UPS	ubiquitin proteasome system
TEM	transmission electron microscopy
ThT	thioflavin T
TRO	trodusquemine
